# Progress in quantitative single-molecule localization microscopy

**DOI:** 10.1007/s00418-014-1217-y

**Published:** 2014-04-20

**Authors:** H. Deschout, A. Shivanandan, P. Annibale, M. Scarselli, A. Radenovic

**Affiliations:** 1 Laboratory of Nanoscale Biology, Institute of Bioengineering, School of Engineering, EPFL, Lausanne, Switzerland; 2 Present Address: Biomedical Engineering Department, University of California, Irvine, CA USA; 3 Present Address: Department of Translational Research and New Technologies in Medicine and Surgery, University of Pisa, Pisa, Italy

**Keywords:** Single-molecule localization microscopy (SMLM), Photoactivated localization microscopy (PALM), Fluorescent protein, Co-localization, Single-molecule counting, Quantitative microscopy, Cluster analysis

## Abstract

With the advent of single-molecule localization microscopy (SMLM) techniques, intracellular proteins can be imaged at unprecedented resolution with high specificity and contrast. These techniques can lead to a better understanding of cell functioning, as they allow, among other applications, counting the number of molecules of a protein specie in a single cell, studying the heterogeneity in protein spatial organization, and probing the spatial interactions between different protein species. However, the use of these techniques for accurate quantitative measurements requires corrections for multiple inherent sources of error, including: overcounting due to multiple localizations of a single fluorophore (i.e., photoblinking), undercounting caused by incomplete photoconversion, uncertainty in the localization of single molecules, sample drift during the long imaging time, and inaccurate image registration in the case of dual-color imaging. In this paper, we review recent efforts that address some of these sources of error in quantitative SMLM and give examples in the context of photoactivated localization microscopy (PALM).

## Introduction

With the invention of single-molecule localization microscopy (SMLM) techniques (Betzig et al. 2006; Hess et al. 2006; Rust et al. 2006), it has become possible to image intracellular proteins with high contrast at a hitherto unprecedented resolution in conditions that resemble their natural environment. Nowadays, SMLM is starting to be used routinely for imaging of biological samples in 2D and 3D, in fixed and live cells, and in multiple colors (Klein et al. 2014; Oddone et al. 2014).

SMLM techniques can be used for quantitative studies, e.g., counting proteins in a single cell, analyzing the spatial organization of proteins, or estimating co-localization between organelles that are smaller than the optical diffraction limit or even between single molecules. SMLM can also be used for other types of quantitative measurements, for instance in single-particle tracking (SPT) mode (Manley et al. 2008; Persson et al. 2013). The high labeling specificity offered by fusion proteins, and the relatively low chance of overcounting caused by repeated imaging of the same fluorophore due to the phenomenon of photoblinking, makes photoactivated localization microscopy (PALM), among the different SMLM techniques, a relatively better choice for quantitative imaging.

However, to use SMLM/PALM for quantitative measurements, a number of issues have to be overcome. Since these techniques provide localizations of individual fluorescent molecules rather than a single image, the tools required for quantitative analysis are often different from these in conventional fluorescence microscopy. Also, imaging with PALM involves multiple sources of errors, such as: overcounting of commonly used fluorescent proteins in the range of 100 % due to photoblinking (Annibale et al. 2010; Lee et al. 2012); limited detection efficiency in the range of 40–60 % related to incomplete photoconversion (Annibale et al. 2012; Durisic et al. 2014); uncertainty in the localization of molecules in the order of 15–50 nm caused by, among other factors, a limited number of detected photons (Mortensen et al. 2010; Thompson et al. 2002); and sample drift during the long imaging time in the order of 50 nm (Betzig et al. 2006). In the case of co-localization analysis using PALM, additional challenges exist in the form of the limited number of available spectrally separate fluorescent proteins for multi-color imaging, and that of accurately overlaying the images from the two-color channels (Annibale 2012). It must also be mentioned that the computational methods used in SMLM, i.e., the image processing and localization algorithms, can be another source of error in quantification (Deschout et al. 2014; Small and Stahlheber 2014).

Here, we review the recently reported efforts toward solving some of the problems that affect quantitative SMLM measurements. In particular, we focus on PALM and its commonly reported applications: counting single molecules, analyzing protein organization, and measuring co-localization on the single-molecule level.

## Single-molecule counting with PALM

Several important cellular functions involve low-copy number proteins that are not detectable by conventional measurement techniques (Ghaemmaghami et al. 2003). Also, studies dealing with the stochastic nature of gene expression and its importance in biology (Elowitz et al. 2002; Raj and van Oudenaarden 2008) require accurate and precise single-molecule counting. While omics-scale abundance data with single-molecule sensitivity can be obtained from conventional fluorescence microscopy (Taniguchi et al. 2010), the spatial resolution is limited due to the diffraction of light. PALM, with the possibility of single-molecule resolution counting in sub-diffraction limit voxels, therefore clearly offers interesting prospects in this field.

In order to use PALM for counting, the ideal scenario would be that each fluorescent protein present is counted once and only once. However, there are at least two critical issues that result in counting errors—undercounting due to a limited detection efficiency and overcounting due to multiple appearances of the same fluorophore. Due to the limited detection efficiency inherent to fluorescence microscopy, resulting from misfolding and incomplete maturation of the fluorescent proteins, only a fraction of the molecules can be imaged. In conventional fluorescence microscopy, this fraction is about 80 % for GFP (Ulbrich and Isacoff 2007). In PALM, even lower fractions are observed, because of the limited photoconversion efficiency. A fraction of 53–60 % has been reported for the relatively bright mEos2 (Annibale et al. 2012; Durisic et al. 2014), and many other fluorescent proteins perform even worse.

Various methods have been developed to work around this obvious limitation. Diffraction-limited protein subunit stoichiometry estimation can be performed by observing the bleaching steps of individual fluorophores attached to the subunit molecules. This method was used to estimate the subunit stoichiometry of membrane proteins (specifically, NMDA receptors) in live cells, composed of two different subunits, by means of labeling with GFP (Ulbrich and Isacoff 2007). The detection efficiency of GFP was estimated by fitting the observed number of bleaching steps to a binomial model for detection. A similar approach was used to estimate the subunit stoichiometry of heteromeric glycine-gated channels (GlyRs) (Durisic et al. 2012). In the context of SMLM, the stoichiometry of the asialoglycoprotein receptor complex in rat hepatic lectin 1 (RHL1) and rat hepatic lectin 2 (RHL2) was estimated by single-molecule counting (Renz et al. 2012). The problem of limited detection efficiency was avoided by focusing on the *ratio* of detected molecules. First, the relative detection efficiency of paGFP/paCherry was characterized, by performing dual-color PALM on a 1:1 fusion construct. Subsequently, dual-color PALM was used to investigate the homo/hetero-association, by determining the ratio between the counts of paGFP-RHL1/paCherry-RHL1 pairs and paGFP-RHL1/paCherry-RHL2 pairs, respectively. In another work, the expected ratio between monomers and dimers of pamCherry1 for a given detection efficiency was modeled, and was fit to data to estimate the detection efficiency (Li et al. 2013).

**Fig. 1:**
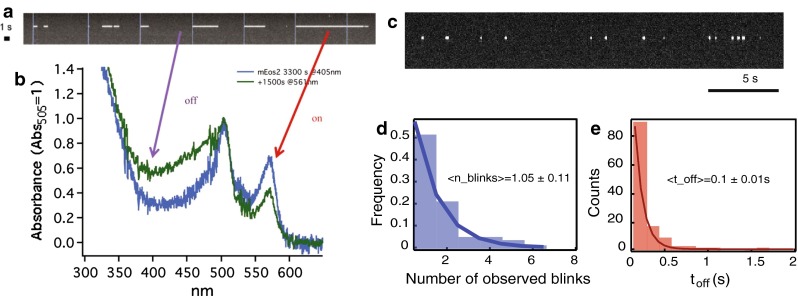
**a** A single-molecule kymograph of an individual mEos2 molecule, upon pulsed 405 nm irradiation (*blue vertical lines*). Taken from Annibale 2012. **b** The spectral evolution of partially photoconverted mEos2 upon 561 nm irradiation, displaying an increase in 405 nm absorbance, corresponding to the protonated form of the *red fluorescent* state. Taken from Annibale 2012. **c** The photoblinking phenomenon exists even at continuous activation. A typical kymograph of an mEos2 molecule embedded in a polymer gel, upon continous 405 nm irradiation at low intensity. Taken from Annibale et al. 2011. **d** A histogram of the number of times a single mEos2 molecule undergoes photoblinking (*n*_blink_) before definitive photobleaching. Experimental values based on a single exponential best fit are shown, the 1/e decay values indicate a mean of *n*_blink_ = 1.05 ± 0.11. Taken from Annibale et al. 2011. **e** A histogram of the measured dark times showing a mean of *t*_off_ = 0.10 ± 0.01 s. Taken from Annibale et al. 2011

**Fig. 2:**
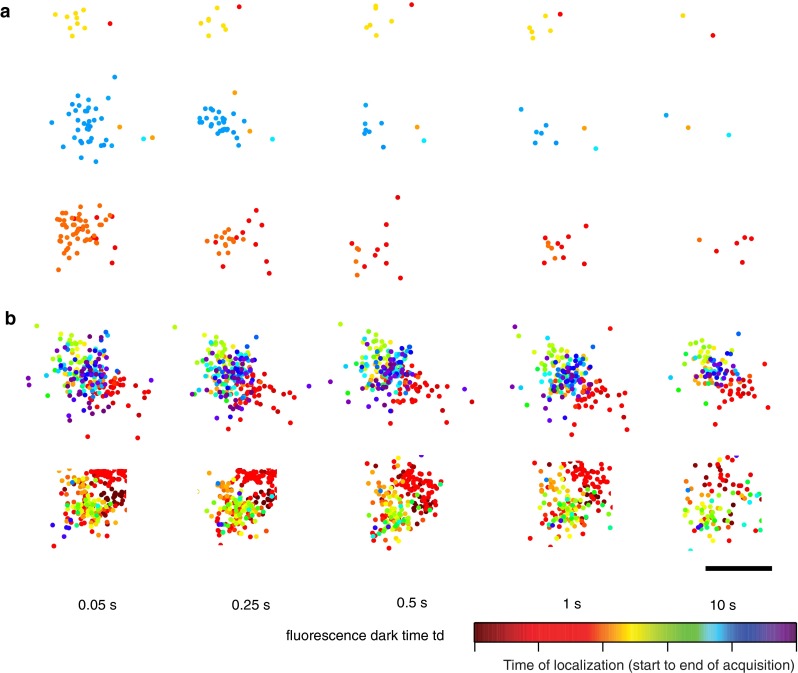
Snapshots of clusters formed by localizations of membrane proteins in fixed HeLa cells. Markers represent single-molecule localizations and their *color* represents the time of localization. **a** Representative images of three artifact spatial-temporal clusters of SrcN15-mEos2 (a negative control for clustering) and their evolution for increasing values of the allowed fluorescence dark time threshold *t*_d_. **b** Representative images of two β2-mEos2 clusters and their evolution with the fluorescence dark time threshold *t*_d_. A temporal artifact component (*red sub*-*cluster*) is also visible in the second cluster. The estimated location of the molecules changes slightly from one *t*_d_ value to another since the number of collected photons and their spatial distribution attributed to each localized molecule changes. *Scale* 100 nm. Taken from Annibale et al. 2011

**Fig. 3:**
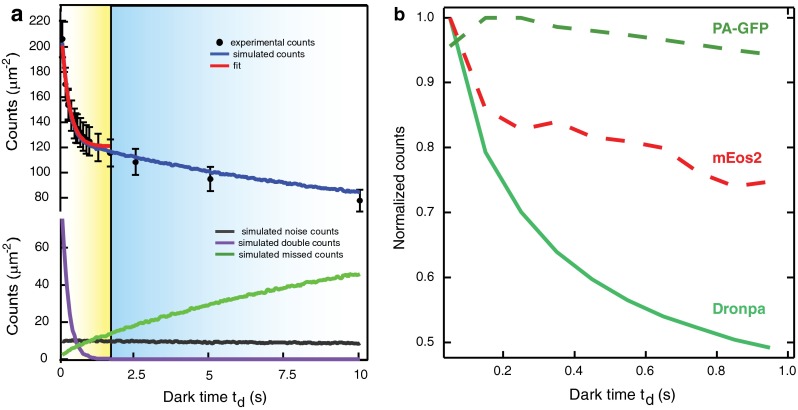
**a** The experimental (*markers*) and simulated (*blue curve*) counts of mEos2 molecules localized as a function of the dark time threshold *t*_d_, together with the simulated counts ascribed to missed counts (*green*), multiple counts (*violet*), and noise (*black*). For all samples, the duration of the acquisition is 20,000 frames × 50 ms. The *red curve* shows the best fit to the data for *t*_d_ values between 0.05 and 2 s. If no missed counts were to occur, the asymptote of the decaying curve of the observed counts would converge to the effective number of molecules present in the sample. Fitting to a negative exponential model yielded a mean of *t*_off_ = 0.260 s and a mean of *n*_blink_ = 0.760, consistent with what was shown in Fig. 1d and e respectively. The fit yielded *N* = 121 ± 6 molecules/µm^2^, whereas the total density of the simulated sample was 135 molecules/µm^2^ including noise counts, resulting in a 10 % error. Taken from Annibale et al. 2011. **b** Comparison of the normalized estimates for counts of localized molecules as a function of *t*_d_, for three different fluorescent proteins: paGFP, Dronpa and mEos2. Taken from Annibale et al. 2011

Another phenomenon that critically affects counting in SMLM is that of overcounting due to photoblinking. In one of the first quantitative studies involving PALM, the photoblinking behavior of the fluorophore (i.e., tdEos) was not taken into account (Greenfield et al. 2009). In the case of its monomeric form mEos2, the phenomenon of photoblinking was investigated by systematic inspection of the fluorophore traces of immobilized molecules in polymer gels (Annibale et al. 2010). Similar to the long-lived dark state of GFP (Dickson et al. 1997), it was found that the activated and excited mEos2 (i.e., on-state) might reversibly go to a long-lived dark state instead of getting photobleached, and later come back to the bright state, as illustrated in Fig. 1a–c. This means that, due to this “blinking” phenomenon, the same molecule might be counted multiple times by a localization algorithm that does not correct for it. In vitro experiments on gels showed that roughly half of the mEos2 molecules are reactivated at least once (Fig. 1d), making it possible that the molecules are overcounted by a factor of 2. In the case of paGFP, the number of reactivations is lower, and for a photoswitchable fluorescent protein such as Dronpa, the number is higher, as shown in Fig. 3b (Annibale et al. 2010). Similar photophysical behavior has been reported for the photoconvertible fluorescent protein mMaple (McEvoy et al. 2012). Since the time spent in the dark state (*t*_off_) is orders of magnitude lower than the duration of the experiment, photoblinking will form small clusters in a time series plot of the localizations for the whole duration of the experiment, as illustrated Fig. 2. This immediately suggests a method to account for photoblinking: by using a threshold in time (*t*_d_) and in space, it is possible to partition these traces in spatial–temporal clusters, and to assign each cluster to one molecule. This apparently simple method was found to be highly effective in correcting for photoblinking (Fig. 2) (Annibale et al. 2011).

How to select the optimal *t*_d_? By collecting the localizations within a set spatial radius that depends on labeling density and localization uncertainty, and within a time interval *t*_d_, and counting them as one localization after performing weighted averaging, it is possible to compute the number of molecules *N*(*t*_d_) counted for different values of *t*_d_. It was found that the empirical *N*(*t*_d_) curve obtained in this way fits well to a negative exponential function, as shown in Fig. 3a. That is, for larger values of *t*_d_, the improvement in counting accuracy becomes asymptotically lower. Also, setting a too high value for this parameter might result in missed localizations, i.e., localizations corresponding to different molecules getting grouped together as one. Therefore, depending on the nature of the application, the value of *t*_d_ should be selected so as to minimize the errors coming from both the multiple counting of a photoblinking molecule and the missed localizations, or a conservative value of *t*_d_ should be chosen so that the observed count is a lower bound, see Fig. 3a (Annibale et al. 2011).

Lee et al. introduced a more detailed model for *N*(*t*_d_), and, based on its photobleaching and blinking behavior, proposed Dendra2 as a better alternative to mEos2 for counting purposes (Lee et al. 2012). Additionally, an imaging strategy called Fermi photoactivation was proposed, which improves the temporal separation in the activation of different molecules, thus helping to overcome undercounting due to the overlapping of molecules in the initial frames of imaging, which might occur when using a fixed activation power during the whole imaging time (Lee et al. 2012). On the other hand, by assuming that the probability of activating a molecule remains constant over time, a relationship between the cumulative number of localizations and the imaging time was found (Gunzenhäuser et al. 2012). Such a relationship can provide a stopping criterion for imaging, given a target accuracy in counting. The method was applied to imaging the HIV structural protein Gag labeled with tdEos and also with mEos2.

Alternatively, a method based on Kalman filtering has been proposed, in order to scan and group photoblinking molecules (Lando et al. 2012). A very different approach, based on the spatial pair correlation function (PCF), was inspired by the special case of spatial cluster analysis of membrane proteins and utilizes the difference between the spatial signature of the multiple appearances of the same molecule due to photoblinking and that of the true protein clusters (Sengupta et al. 2011). Another method was proposed to estimate the average number of localizations per molecule in samples that form definite spatial structures (e.g., microtubules or actin filaments), mainly in the context of stochastic optical reconstruction microscopy (STORM) (Nieuwenhuizen et al. 2013). This approach, based on Fourier ring correlation analysis, can also be used to estimate the resolution obtained in SMLM images, although only samples with definite spatial structures were investigated. Others have reported a similar measure to estimate the resolution in SMLM (Banterle et al. 2013).

## Quantitative analysis of heterogeneity in protein spatial organization

One of the niche areas in cell imaging that SMLM appeals to is the study of spatial heterogeneity in protein organization; e.g., that of membrane proteins appearing as micro- or nanodomains rather than individual molecules diffusing freely along the membrane, and its function in, for instance, signaling. In addition to membrane proteins, other systems with spatial heterogeneity can also be studied with SMLM. Indeed, SMLM has been used to study protein spatial organization in various systems including signaling receptors in the *Escherichia Coli* chemotaxis signaling network (Greenfield et al. 2009), signaling proteins in T-cells (Rossy et al. 2013; Williamson et al. 2011), GPI-anchored proteins (Sengupta et al. 2011), G protein-coupled receptors (GPCRs) (Scarselli et al. 2012, 2013), SNAP receptor (SNARE) complexes (Pertsinidis et al. 2013), and RNAP in *E. Coli* (Endesfelder et al. 2013). While most of these studies have focused on the characterization of heterogeneity in spatial organization and its dependence on different conditions, some have even used the estimated parameters to fit biophysical models (Greenfield et al. 2009; Hess et al. 2007). A brief discussion of some of the questions and studies in this field can be found elsewhere (Lang and Rizzoli 2010; Owen and Gaus 2013).

Various clustering and cluster analysis techniques have been used for the analysis of spatial heterogeneity in SMLM images, in particular the quantification of nanodomain properties and their comparison at different conditions. These approaches can be divided into two broad categories: (1) exploratory analysis tools from spatial statistics that have been used for similar problems in electron microscopy (Parton and Hancock 2004; Zhang et al. 2006), such as PCF and Ripley’s *L*(*r*)-*r* function, or the nearest neighbor distance distribution (Endesfelder et al. 2013) and (2) clustering by means of algorithms such as density-based spatial clustering of applications with noise (DBSCAN) (Ester et al. 1996), followed by analysis of the obtained clusters by various methods to estimate cluster parameters, e.g., by averaging or by fitting each cluster to a normal distribution to estimate the cluster radius.

An introduction to the first approach can be found elsewhere (Diggle 2003; Gould et al. 2012). Briefly, the Ripley’s *K*(*r*) function is the ratio of the average number of extra localizations within distance *r* of a randomly chosen point and the density of localization in the area of analysis, and *L*(*r*) is the transformation $\sqrt {\frac{K(r)}{\pi }}$ with certain convenient properties. For instance, *L*(*r*)-*r*, by definition, is equal to zero for a point pattern that is distributed completely at random, i.e., complete spatial randomness (CSR). *L*(*r*)-*r* is greater than zero if the points are clustered and is less than zero if the point pattern shows regularity. The magnitude of *L*(*r*)-*r* is a measure for the degree of clustering and can be used for comparison between different conditions. The value of *r* corresponding to the maximum of *L*(*r*)-*r* gives an estimate of the average cluster radius in the point pattern. The PCF *g*(*r*) is a closely related measure, *K*(*r*) being the integral of $2\pi rg(r)$, and it can also provide estimates of parameters like the ones mentioned above. These measures can also be used to estimate other parameters such as the number of localizations per cluster (Parton and Hancock 2004; Sengupta et al. 2011; Zhang et al. 2006), and the effective potential of the mean force between the localized molecules (Veatch et al. 2012). The *L*(*r*)-*r* function has an advantage when compared to the PCF in that, since *L*(*r*)-*r* is based on an integration over the radius *r*, it is less influenced by noise. On the other hand, this also means it is less sensitive and that systematic errors such as overcounting due to photoblinking are accumulated over *r*. Therefore, when this measure is used, photoblinking artifacts must be accounted for by one of the methods mentioned in the section on counting.

The first approach, i.e., exploratory tools such as PCF or Ripley’s function, has been extended to account for some error sources inherent to SMLM. In a technique called pair correlation PALM (PC-PALM), the PCF approach is extended by means of a model to differentiate the artifact clusters due to fluorophore photoblinking from true proteins clusters (Sengupta et al. 2011). Modifications of the *L*(*r*)-*r* function have been suggested to incorporate membrane curvature characteristics, since 2D imaging of proteins in undulating membranes can cause clustering artifacts (Owen et al. 2013). This work also shows the applicability of Ripley’s function in the case of 3D localization data. While this approach is promising, it uses *L*(*r*)-*r* only to identify clusters (Owen et al. 2010), rather than as an exploratory statistical tool to be used for inference and comparison (Hess et al. 2007; Lillemeier et al. 2010; Scarselli et al. 2012).

The nearest neighbor approach as an exploratory tool involves finding the nearest neighbor distance distribution within a point pattern and comparing it to one that corresponds to a point pattern distributed by CSR. The contrast between the nearest neighbor distance method and correlation methods such as *L*(*r*)-*r* or PCF is that, since the former looks at nearest neighbors only, it focuses on information on the short scale, whereas the latter gives information on a variety of scales.

It should be noted that Ripley’s function and PCF are defined for a stationary, spatially homogeneous point process only, i.e., the average density within the point pattern is assumed to be independent of the spatial location. If the point process is spatially inhomogeneous, e.g., due to a spatial gradient in protein locations, other extensions must be used in order to be statistically more accurate (Baddeley et al. 2000). Also, the inevitable choice of limiting the analysis to a window results in the exclusion of the points near the borders, often resulting in a significantly biased estimation. Various edge correction methods are available to correct for this bias (Haase 1996).

In the case of the second approach, i.e., clustering followed by parameter inference, various algorithms are used for the clustering part. The DBSCAN algorithm is the most popular one (Annibale 2012; Endesfelder et al. 2013; Li et al. 2013; Pertsinidis et al. 2013), although other methods have also been used (Gunzenhäuser et al. 2012; Lelek et al. 2012; Owen et al. 2010). DBSCAN works by exploiting the density difference between clusters and the background, i.e., the density in the neighborhood of a point must exceed a threshold in order to be identified as part of a cluster. This method has several advantages over other commonly available clustering algorithms, including that it does not need an a priori number of clusters to be provided as input, that it can identify clusters of arbitrary shapes, and that it can account for background noise (and for a monomer fraction). An algorithm based on DBSCAN to account for errors in clustering due to the presence of localization uncertainty in PALM was used to study RAF multimer formation and signaling (Li et al. 2013). However, identifying the parameters required by DBSCAN is another problem, which is often solved empirically (Annibale 2012; Endesfelder et al. 2013; Pertsinidis et al. 2013), even though some have used the heuristic suggestions of the original DBSCAN paper on how to set the parameters (Bar-On et al. 2012). Extensions such as OPTICS that do not need these parameters as input might also be useful (Ankerst et al. 1999).

The choice between the two above-mentioned approaches depends on the problem at hand. In general, the first approach (i.e., exploratory tools such as PCF or *L*(*r*)-*r*) is less arbitrary than the second one (i.e., clustering followed by characterization). However, since PCF or Ripley’s function estimate an ensemble parameter, e.g., cluster radius, for the whole area of analysis rather than for individual clusters, they may not be the ideal tool if the parameters show significant variation between clusters. Similar problems might arise if the cluster shapes are elliptical or asymmetric and the study of the shape parameters is important. In such cases, the approach of clustering followed by parameter estimation for individual clusters might be more suitable.

Protein assemblies such as nuclear pore complexes (NPCs) are ideal systems for the application of SMLM, due to their fixed protein stoichiometry and structure. Systematic labeling of different NPC components combined with averaging of thousands of corresponding SMLM images allowed the creation of a human NPC scaffold structure model with a localization uncertainty well below 1 nm (Szymborska et al. 2013). In this study, imaging with both immunolabeling as well as fusion protein/nanobody labeling were done separately, and in the case of many proteins, the former was found to systematically overestimate the NPC radius by around 7 nm (about 15 %), possibly due to the larger size of primary and secondary antibodies. Prior work on NPCs with a similar averaging approach had also achieved major improvements in resolution (Loschberger et al. 2012). Integrated targeted proteomics and PALM were used by Ori et al. to determine the absolute stoichiometry of the NPC, which was found to vary across different human cell lines (Ori et al. 2013).

## Toward quantitative co-localization with dual-color SMLM

Having reviewed in the previous two sections the SMLM-based methods for counting single molecules and investigating protein spatial heterogeneity, we will now discuss the ability of dual-color SMLM to measure co-localization on the single-molecule level. Fluorescence microscopy in general is an excellent tool to probe potential interactions between cellular objects by measuring their co-localization. This requires labeling of the different objects with spectrally separate fluorophores and subsequent recording of an image in each of the corresponding color channels. The co-localization between the objects can then be visualized by simply overlaying the images. Quantification is also possible, for instance, by estimating the correlation between the pixel values in the overlaid images (Bolte and Cordelieres 2006; Dunn et al. 2011; Zinchuk et al. 2007). While the resolution in diffraction-limited microscopy usually restricts the interpretation of co-localization to the level of organelles or other objects of similar size, far more detailed information is offered by SMLM. In theory, these techniques even allow to investigate the co-localization between individual molecules. As a consequence, SMLM techniques are already being embraced by biologists that aim to unravel the mechanisms that govern protein–protein interactions (Lehmann et al. 2011; Lubeck and Cai 2012; Sherman et al. 2011; Winckler et al. 2013). In the following sections, we will review the practical problems that are present in using dual-color SMLM for quantitative experiments and discuss the recent approaches to solve those problems.

### Image registration

**Fig. 4:**
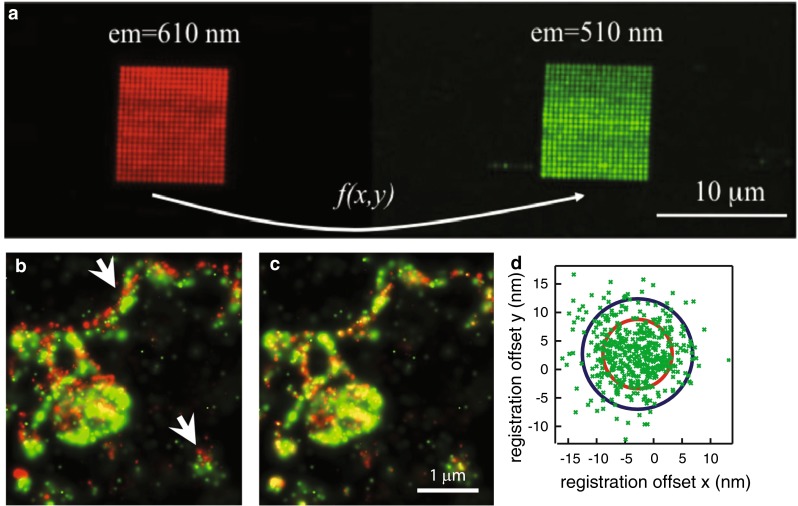
An illustration of the image registration procedure to align images from different color channels. **a** An image obtained by integrating the images in the *green* and *red* channel of a fiducial scanned across a square grid with a size of ~10 µm. **b** An overlay of the *red* and *green* PALM images of a membrane patch of a cell that expressed the protein SrcN labeled with a fusion construct of psCFP2 and mEos2, prior to image registration and **c **after image registration. **d** A scatter plot of the residual offset *x*_g_ − *x*_r_ and *y*_g_ − *y*_r_, with *x*_g_ and *x*_r_ being the *x*-coordinates in the *green* and *red* channel respectively, and *y*_g_ and *y*_r_ being the *y*-coordinates in the *green* and *red* channel respectively. The *blue circle* has a radius of 10 nm, the *red circle* has a radius given by $\sqrt {\sigma_{\text{g}}^{2} + \sigma_{\text{r}}^{2} + {\text{TRE}}}$, with *σ*_g_ and *σ*_r_ being the localization precisions in the *green* and *red* channel respectively. The residuals were extracted from the trajectory of a fluorescent bead with 100 nm diameter, immobilized on the coverslip and imaged during a time lapse movie. Adapted from Annibale 2012 and Annibale et al. 2012

One key requirement for co-localization analysis is a sufficiently precise overlay of the images in the different color channels. This is especially challenging for SMLM-based co-localization, since the images are rendered from single fluorophore locations that are usually determined with an uncertainty in the order of 15–50 nm (Mortensen et al. 2010; Thompson et al. 2002). The procedure for aligning the images, i.e., the image registration, starts with localizing fiducials that are visible in the different color channels. This results in a list of positions for each color channel that should be identical after alignment, allowing to estimate a function that maps one channel onto another one (Goshtasby 1988). Different types of fiducials have been reported, such as a lattice that contains optical holes in a grid with known spacing (Koyama-Honda et al. 2005; Pertsinidis et al. 2013) or a geometrical structure inside the sample itself, such as the center of the ring-shaped nuclear pore complex (Loschberger et al. 2012). A more popular type of fiducials that do not require special manufacturing or prior knowledge of the sample are beads that are fluorescent in both color channels (Baddeley et al. 2011; Bates et al. 2012; Churchman et al. 2005; Lehmann et al. 2011). In order to illustrate the importance of image registration, dual-color PALM was performed on a fusion construct of psCFP2 and mEos2 attached to the cell membrane protein SrcN (Annibale 2012). An isolated bead was used as a fiducial, and it was moved in the field of view along a grid pattern, using a piezo stage, and at each grid position an image was recorded in both color channels, as illustrated in Fig. 4a. It is clear that a correct overlay was obtained only after image registration, as can be seen in Fig. 4b and c.

In order to properly interpret the measured co-localization, it is necessary to quantify the precision of the image registration procedure. One often used measure for this precision is the target registration error (TRE), which can be interpreted as the mean offset between the positions of the fiducials in both color channels after image registration (Churchman et al. 2005; Cohen and Ober 2013). TRE values below 10 nm are typically reported (Annibale et al. 2012; Bates et al. 2012; Churchman et al. 2005; Malkusch et al. 2012; Pertsinidis et al. 2013), and one study even achieved a TRE below 1 nm within a single pixel, by accounting for pixel response non-uniformities and mechanically stabilizing the microscope with an active feedback system (Pertsinidis et al. 2010). The evolution of the image registration precision over time was investigated by recording a time lapse movie of a bead, while using an axial stability feedback system (Annibale et al. 2012). During acquisition, the bead followed a trajectory determined by the lateral drift of the setup. While the TRE was 4.5 nm, the mean of the differences between the positions of the bead between both color channels after registration had a larger value of 6.7 nm, possibly due to long-term mechanical instabilities, as shown in Fig. 4d.

### Fluorescent protein pairs

If the detection efficiency for the label in one channel is *x*, and that in the other channel is *y*, then the estimated co-localization underestimates the true co-localization by a factor *xy*, assuming a linear relation to the co-localization measure used. In other words, the correct estimation of co-localization in dual-color PALM experiments is possible only if the fraction of fluorescent proteins that did not photoconvert to the on-state is accounted for. Several investigations have been undertaken to measure the photoconversion efficiency of different fluorescent proteins, for instance, by monitoring the change in the absorbance spectrum of a solution upon irradiation with 405 nm light (Annibale et al. 2012; Wiedenmann et al. 2004). However, the photoconversion efficiency of a fluorescent protein in this in vitro environment might be altered with respect to the cellular environment. One recent study has, therefore, attempted to measure the photoconversion efficiency inside cells, by counting either the photoconversion or the photobleaching events corresponding to individual fluorescent proteins that are attached to the subunits of the cell membrane receptor GlyR (Durisic et al. 2014). Among several other fluorophores, they found a photoconversion efficiency of ~60 % for mEos2 and ~50 % for pamCherry. Multiplication of these values can be used as an estimate of the efficiency with which the co-localization between the corresponding fluorophores can be observed.

However, such an estimate might not reflect the true co-localization efficiency, as it is determined from single-color PALM experiments, while the illumination procedure in a dual-color PALM experiment can increase the rate at which fluorescent proteins photobleach before being photoconverted to the on-state. Dual-color PALM experiments performed on 1:1 fusion constructs of both fluorescent proteins, for instance inside a polymer gel or attached to a membrane protein, provide a solution (Annibale et al. 2012; Renz et al. 2012). Since one observes the same fluorophore pattern in both color channels, the measured fraction of co-localized fluorophores provides an alternative estimate of the co-localization efficiency. This fraction was measured for fusion constructs of three pairs, namely: psCFP2-pamCherry, Dronpa-mEos2 and psCFP2-mEos2 (Annibale et al. 2012). For the latter pair, virtually no co-localization was found, probably due to photobleaching of psCFP2 during activation of mEos2. The other two pairs gave rise to a ~15 % co-localization fraction, which can partially be explained by the photoconversion efficiencies of the fluorophores. Although mEos2 has a superior photon yield, psCFP2 and pamCherry are arguably the most suitable pair for dual-color PALM, since pamCherry is not fluorescent in the off-state and therefore allows simultaneous image acquisition in both color channels.

### Co-localization analysis

The output of an SMLM experiment can be represented as a pixelated image, for instance, by giving each pixel a value that scales linearly with the number of localized fluorophores inside the area that corresponds to that pixel. This means that intensity-based co-localization methods that rely on quantifying the correlation between the pixel values of images in different color channels (Bolte and Cordelieres 2006; Dunn et al. 2011; Zinchuk et al. 2007) can in principle be applied. However, such correlations are challenging to interpret, as they are highly susceptible to overestimation caused by noise and bleed-through (Bolte and Cordelieres 2006). One recent study reports a method that allows correcting for bleed-through in the context of SMLM (Kim et al. 2013).

**Fig. 5:**
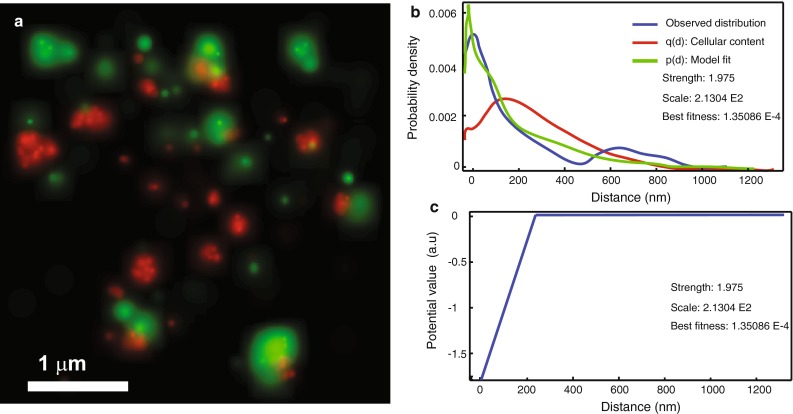
Spatial interaction analysis of dual-color PALM images. The method uses a nearest neighbor spatial interaction model based on Gibbs statistics, which characterizes an interaction by means of a potential (Helmuth et al. 2010). **a** Dual-color PALM data represented as probability maps. The *green* channel shows the GPCR protein β2-adrenergic receptor labeled with psCFP2, and the *red* channel shows clathrin light chain labeled with pamCherry (Annibale 2012; Annibale et al. 2012). **b** Results of interaction analysis: the observed nearest neighbor distance distribution between the two channels (*blue*); the result of fitting the spatial interaction model with a linear L1 potential to this distribution (*green*); the curve corresponding to the null hypothesis of “no interaction”, estimated by accounting for the intra-point pattern distance distribution (*red*). The method also returns the inferred parameters (i.e., strength and scale) that can be used for comparison. **c** The inferred interaction potential. Adapted from Shivanandan et al. 2013

Since raw SMLM data consist of locations of individual fluorophores, object-based co-localization methods (Bolte and Cordelieres 2006) can be used without any prior data processing. Usually, co-localization between objects in different color channels is investigated by calculating the distance between their positions and comparing it to a predefined threshold. However, it is challenging to define an optimal value for this threshold, and sometimes a rather arbitrary value of ~200 nm based on the diffraction-limited resolution is used. An object-based method was, therefore, recently developed that can estimate the threshold value from the data, by modeling the nearest neighbor distance distribution in a spatial statistics framework that estimates a spatial interaction potential between the objects in the different color channels (Helmuth et al. 2010). In addition to this feature, the method also extends the classical threshold-based co-localization by providing other interaction “potentials” apart from the threshold function, and also incorporates in the model the spatial distribution of objects within a point pattern. The latter corrects for the fact that estimates of spatial interaction, e.g., co-localization, depend on the intra-object spatial distribution. This method was found to be robust against errors in the identification of the objects by image processing. In the context of PALM, this method was applied to investigate the co-localization between pamCherry-labeled clathrin-coated vesicles and psCFP2-labeled GPCRs during internalization (Shivanandan et al. 2013), as illustrated in Fig. 5. Another solution for the dependency of co-localization on the intra-object distribution has recently been reported by taking into account the spatial distribution of the objects (Malkusch et al. 2012). This object-based method has the extra advantage that it corrects for photoblinking. Another approach that is frequently reported in the context of SMLM-based co-localization is the spatial cross-correlation analysis which uses the bivariate version of the PCF, called the cross-correlation function (CCF) (Gunewardene et al. 2011; Pertsinidis et al. 2013; Sengupta et al. 2011; Veatch et al. 2012).

## Conclusion and outlook

We have reviewed recent developments in SMLM for counting single molecules, analyzing the heterogeneity of the spatial distribution of proteins and measuring co-localization on the single-molecule level. As quantitative SMLM-based methods for these purposes have only recently been reported, there are still several problems and difficulties that need to be addressed. For instance, any study that uses SMLM for quantitative analysis must have stringent negative and positive controls, since artifacts in the imaging or analysis methods can give rise to wrong inferences. Also, the data must be corrected for sample drift by means of fiducial markers, or by correlative or statistical approaches based on the data itself (Geisler et al. 2012; Mlodzianoski et al. 2011). Working with the localizations directly rather than image representations such as histograms or probability maps is better for quantitative analysis, as the latter involves a loss of information. A challenge remains in identifying well-accepted standard methods for the quantitative analysis of SMLM, which would allow researchers to perform the correct comparison between reported results.

An important issue, especially in analyzing the spatial heterogeneity or co-localization of proteins, is the effect of localization uncertainty (Deschout et al. 2014; Mortensen et al. 2010; Thompson et al. 2002). Not incorporating this effect into the analysis might result in incorrect estimates. For instance, in the case of cluster analysis, the presence of localization uncertainty, equivalent to sampling from a circular or elliptical Gaussian distribution (Thompson et al. 2002), will result in deformed if not enlarged clusters being imaged. Also, the uncertainty in position estimates results in an uncertainty in distances computed from them and hence affects object-based co-localization (Ruprecht et al. 2010). Measures that do not account for the localization uncertainty might result in a wrong interpretation in both cases. The PC-PALM technique that accounts for photoblinking artifacts also incorporates a localization uncertainty model in the analysis, but only through the average uncertainty of all molecule localizations, and its effect was not studied systematically. Defining a cutoff value for the localization uncertainty distribution to select only the more precise molecular localizations can result in artifacts, especially if the localization uncertainty is not homogeneously distributed in space. This problem was investigated in the case of the CCF, which is used to study inter-protein interactions in dual-color PALM, from a purely empirical perspective, with rather mixed results (Sherman et al. 2013).

Besides accounting for the localization uncertainty, progress is required on other issues as well in order to achieve quantitative co-localization on the single-molecule level. The community would benefit from a uniform measure of the registration error, allowing comparison between co-localization results from different studies. An important limitation toward single-molecule level co-localization in the context of PALM is the low co-localization efficiency of current fluorescent protein pairs, necessitating the search for more promising candidates (Bourgeois and Adam 2012).

Artifacts in the sample can also pose challenges to quantitative SMLM. Many of the studies reported in this review were done on fixed samples, although it has been observed that fixation can introduce artifacts in the protein spatial configuration (Annibale et al. 2012; Tanaka et al. 2010). A rigorous investigation of different fixation techniques will therefore be helpful. Also, it has been noticed that SMLM images of organelles such as mitochondria (Betzig et al. 2006), microtubules, or clathrin-coated pits have localization densities that are spatially inhomogeneous, often resulting in spurious structures, e.g., clathrin-coated pits with poor symmetry (Lang and Rizzoli 2010). It will be useful to study this phenomenon in more detail, perhaps by means of correlative microscopy, i.e., by imaging the same structure with other high-resolution imaging techniques such as transmission electron microscopy (TEM) or atomic force microscopy (AFM). Such studies might also provide validations about localization uncertainty and detection efficiency.
